# Limbic encephalitis: Experience of a moroccan center

**DOI:** 10.1002/brb3.1177

**Published:** 2018-11-25

**Authors:** Moussa Toudou‐Daouda, Ahmed Filali‐Adib, Aicha Slassi, Mohammed‐Faouzi Belahsen, Zouhayr Souirti

**Affiliations:** ^1^ Department of Neurology National Hospital of Niamey Niamey Niger; ^2^ Department of Neurology Hassan II University Teaching Hospital Fez Morocco; ^3^ Laboratory of Epidemiology, Clinical Research and Health Community Faculty of Medicine and Pharmacy Sidi Mohammed Ben Abdallah University Fez Morocco; ^4^ Clinical Neuroscience Laboratory, Faculty of Medicine and Pharmacy Sidi Mohamed Ben Abdellah University Fez Morocco; ^5^ Sleep Medicine Center Hassan II University Teaching Hospital Fez Morocco

**Keywords:** autoimmune limbic encephalitis, herpes, limbic encephalitis, sarcoidosis, syphilis, tuberculosis, varicella

## Abstract

**Objectives:**

Histologically defined as an inflammation—degeneration of limbic structures, limbic encephalitis (LE) is a rare disease and often difficult to diagnose particularly in institutions with limited access to laboratory tests such as antineuronal antibodies or HSV‐PCR, and functional imaging. We aimed to describe the demographic, clinical, paraclinical, and etiological features of LE, as well as its medium‐term prognosis in Moroccan patients.

**Materials and Methods:**

We collected retrospectively all patients diagnosed with LE in the Department of Neurology of the University Hospital Hassan II of Fez (Morocco) between September 2008 and December 2016. We analyzed their demographic features, clinical manifestations, magnetic resonance imaging and laboratory findings, etiologies, and medium‐term prognoses.

**Results:**

We included 22 men and 9 women aged 14–76 years (mean age: 45.8 years). In 64.5% of cases, the onset of symptoms was acute. The clinical manifestations included generalized status epilepticus (16.1%), confusional syndrome (29%), epileptic seizures (38.7%), psychiatric disorders (48.4%), and memory disorders (45.2%). The nonlimbic symptoms were nuchal stiffness (22.6%), headaches (9.7%), fever (61.3%), vesicular rash (3.2%), and language disorders (6.5%). The different etiologies found were herpes simplex virus (6.5%), syphilis (16.1%), tuberculosis (3.2%), varicella (3.2%), paraneoplastic autoimmune LE (22.6%), anti‐NMDA‐R LE (6.5%), and sarcoidosis (3.2%). We found 12 cases (38.7%) of LE without definite etiology and with an incomplete diagnostic workup. The medium‐term clinical course includes a complete remission in 45.2% of cases and partial remission in 45.1% of cases. The different sequelae were temporal lobe epilepsy (9.7%), anterograde amnesia (16.1%), and severe cognitive impairment (19.4%). The mortality rate was 9.7% (3 patients).

**Conclusion:**

Our study shows a wide diversity of etiologies of LE in Morocco with essentially an acute mode of onset of symptoms.

## 1 INTRODUCTION

Histologically defined as an inflammation—degeneration of limbic structures (Brierley, Corsellis, Hierons, & Nevin, 1960; Corsellis, Goldberg, & Norton, 1968), limbic encephalitis (LE) is a rare disease and often difficult to diagnose particularly in institutions with limited access to functional imaging and laboratory tests such as herpes simplex virus polymerase chain reaction (HSV‐PCR) and antineuronal antibodies. The clinical manifestations of LE are diverse, and the most common are short‐term memory disorders, psychiatric disorders, confusional state, and temporal lobe epilepsy, which have an acute or subacute onset (Anderson, & Barber, 2008; Corsellis et al., 1968; Geisler et al., 2013; Kerling, Blümcke, & Stefan, 2008). The diagnosis of LE based on both clinical manifestations that suggest the involvement or dysfunction of the limbic system, magnetic resonance imaging (MRI) or functional imaging [SPECT (single‐photon emission computed tomography) or FDG‐PET (fluorodeoxyglucose‐positron emission tomography)] findings and laboratory findings (Asztely, & Kumlien, 2012). Etiologies of LE are multiple, and the most common are infectious causes and autoimmune encephalitis (Asztely, & Kumlien, 2012; Fujimoto et al., 2001).

The aim of our study was to describe the demographic, clinical, paraclinical, and etiological features of LE, as well as its medium‐term prognosis in Moroccan patients.

## 2 METHODS

We collected retrospectively all patients diagnosed with LE in the Department of Neurology of the Hassan II University Teaching Hospital of Fez (Morocco) between September 2008 and December 2016. We collected all the data from the medical records of the patients from the archives of our Department. All our patients had a suggestive clinical picture of acute or subacute encephalitis associating to various degrees the following symptoms: short‐term memory disorders, confusional state, epileptic seizures, and behavior disorders. We selected only patients with positive brain MRI (signal abnormalities in the limbic structures on T2‐weighted fluid‐attenuated inversion recovery [FLAIR] imaging and T2‐weighted images) due to limited access in Morocco to functional imaging and laboratory tests such as HSV‐PCR and antineuronal antibodies that could help to establish a diagnosis of LE with negative brain MRI. We analyzed demographic features, clinical manifestations, MRI and laboratory findings, etiologies, and medium‐term prognosis of all patients included in the study. All patients received a routine cerebrospinal fluid (CSF) examination. Serological tests for syphilis in the blood and CSF were performed in all patients (except in one patient). Some patients received HSV‐PCR in CSF (essentially in the case of acute onset of symptoms), systemic immunological tests (soluble antinuclear antigen antibodies, antinuclear antibodies, anti‐double‐stranded DNA), thyroid function tests (triiodothyronine [T3], thyroxine [T4], thyroid‐stimulating hormone [TSH], anti‐TSH receptor antibodies, antithyroperoxidase, antithyroglobulin), measurement of antineuronal antibodies in blood and or CSF by an indirect immunofluorescence assay (anti‐Hu, anti‐Yo, anti‐CV2, anti‐Ma2, antiamphiphysin, anti‐NMDA‐R [*N*‐methyl‐D‐aspartate receptor], anti‐LGI1 [leucine‐rich glioma inactivated 1], anti‐CASPR2 [contactin‐associated protein 2], anti‐AMPA‐R [alpha‐amino‐3‐hydroxy‐5‐methyl‐4‐isoxazole propionic acid receptor], anti‐GAD [glutamic acid decarboxylase]), thoracic‐abdominopelvic CT scan, and pelvic ultrasound.

This study was approved by the Ethics Committee of Hassan II University Teaching Hospital of Fez (Morocco) in accordance with the Declaration of Helsinki.

## 3 RESULTS

On 3,840 patients hospitalized in our Department during the period of the study (from September 2008 to December 2016), we collected 31 patients diagnosed with LE with a hospital frequency of 0.81%. Table 1 summarizes the demographic, clinical, and etiological features of the 31 Moroccan patients, as well as their medium‐term prognoses. The mean age of the patients was 45.8 years, and 71% were males. The onset of symptoms was acute (1–7 days) in 64.5% of the cases. The clinical manifestations included generalized status epilepticus (16.1%), confusional syndrome (29%), epileptic seizures (38.7%), psychiatric disorders (48.4%), and memory disorders (45.2%). The nonlimbic symptoms were fever (61.3%), nuchal stiffness (22.6%), headaches (9.7%), vesicular rash (3.2%), and language disorders (6.5%).

**Table 1:** Demographic, clinical, and etiological features, as well as the medium‐term prognosis of the 31 patients

Variables	Total (*n* = 31)
Sex	
Males	22 (71%)
Females	9 (29%)
Sex ratio M/F	2.44
Age (year)	
Mean	45.8 ± 15.40
Range	14 and 76
14–24	4 (12.9%)
25–34	4 (12.9%)
35–44	5 (16.1%)
45–54	10 (32.3%)
≥55	8 (25.8%)
Past medical history	
Ovarian teratoma	1 (3.2%)
Melanoma of the thigh	1 (3.2%)
Cancer of the cavum	2 (6.5%)
Arterial hypertension	3 (9.7%)
Diabetes	2 (6.5%)
Hypothyroidism	1 (3.2%)
Myocardial infarction	1 (3.2%)
Thyroid cancer	1 (3.2%)
Thyroidectomy for follicular adenoma	1 (3.2%)
Onset of symptoms	
Acute	20 (64.5%)
Subacute	11 (35.5%)
Limbic symptoms	
Confusional syndrome	9 (29%)
Generalized epilepticus status	5 (16.1%)
Epileptic seizures	12 (38.7%)
Memory disorders	14 (45.2)
Psychiatric disorders	15 (48.4%)
Nonlimbic symptoms and signs	
Vesicular rash	1 (3.2%)
Headache	3 (9.7%)
Fever	19 (61.3%)
Nuchal stiffness	7 (22.6%)
Language disorders	2 (6.5%)
Etiologies	
Syphilis	5 (16.1%)
Herpes	2 (6.5%)
Varicella	1 (3.2%)
Tuberculosis	1 (3.2%)
Sarcoidosis	1 (3.2%)
Paraneoplastic autoimmune limbic encephalitis	7 (22.6%)
Anti‐NMDA receptor limbic encephalitis	2 (6.5%)
Limbic encephalitis with indefinite etiology	12 (38.7%)
Evolution	
Death	3 (9.7%)
Complete remission	14 (45.2%)
Anterograde amnesia	5 (16.1%)
Severe cognitive impairment	6 (19.4%)
Temporal lobe epilepsy	3 (9.7%)

**Figure 1:**
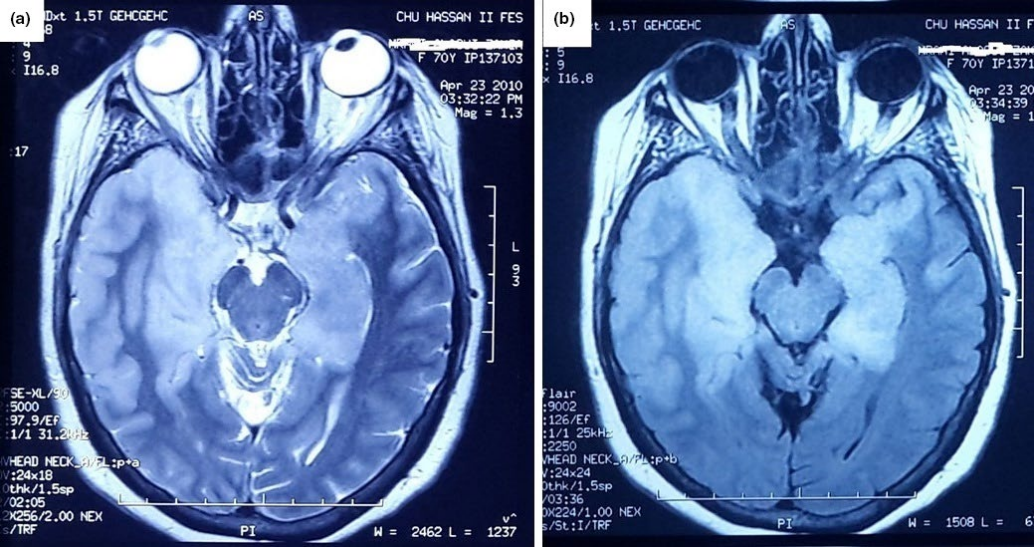
It is a 70‐year‐old woman admitted for a confusional syndrome associated with epileptic seizures, abnormal behavior, and memory disorders. *Brain MRI* shows on FLAIR sequences (a) and T2‐weighted images (b) bilateral hyperintensities in the medial temporal lobes. Thoracic‐abdominopelvic CT scan showed lung cancer. Diagnosis of paraneoplastic autoimmune LE was made after the biological investigations

**Figure 2:**
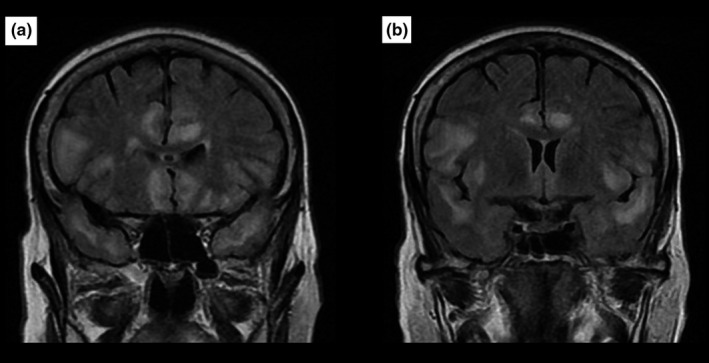
It is a 68‐year‐old woman without no past medical history, admitted for epileptic seizures associated with abnormal behavior and language disorders. *Brain MRI* shows on FLAIR sequences (*a* and *b*) bilateral hyperintensity in the temporo‐insular and cingulate regions. Diagnosis of anti‐NMDA‐R LE was made after the biological investigations

**Figure 3:**
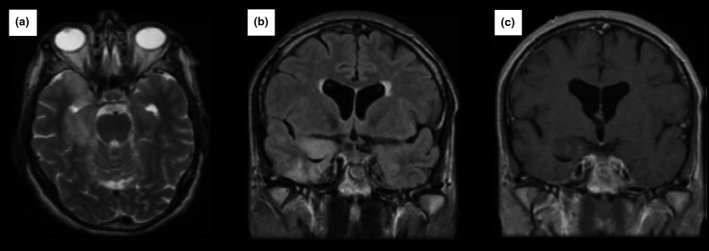
It is a 41‐year‐old man without no past medical history, admitted for generalized epilepticus status. *Brain MRI* shows on T2‐weighted images (a) and FLAIR sequences (b) hyperintensity in the right temporo‐insular without enhancement on gadolinium‐enhanced T1‐weighted images (c). Diagnosis of syphilitic LE was made after the biological investigations

**Figure 4:**
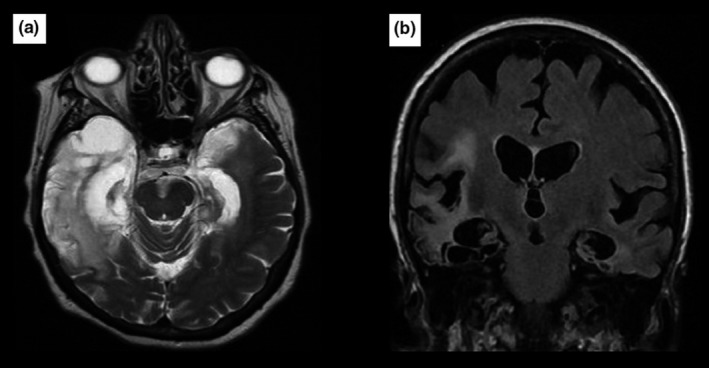
It is a 65‐year‐old man without no past medical history, admitted for the febrile confusional syndrome. *Brain MRI* shows on T2‐weighted images (a) and FLAIR sequences (b) bilateral hyperintensity in temporo‐insular regions predominant on the right. Diagnosis of herpetic LE was made after the biological investigations

The signal abnormalities revealed by brain MRI (Figures 1, 2, 3, 4) and the EEG findings are presented in Table 2.

**Table 2:** MRI and EEG findings

Variables	*n (%)*
MRI	
Bilateral mesiotemporal hyperintensities	12 (38.7)
Unilateral mesiotemporal hyperintensities	1 (3.2)
Bilateral insular hyperintensities	4 (12.9)
Unilateral insular hyperintensities	1 (3.2
Bilateral basifrontal hyperintensities	11 (35.5)
Unilateral basifrontal hyperintensities	4 (12.9)
Bilateral temporo‐insular hyperintensities	13 (41.9)
Unilateral temporo‐insular hyperintensities	4 (12.9)
EEG	
Not done	6 (19.4)
Normal	2 (6.5)
Bilateral slow waves	8 (25.8)
Bilateral spikes and waves	4 (12.9)
Bilateral spikes	3 (9.7)
Slowing of the background rhythm	9 (22.6)
FIRDA/TIRDA	1 (3.2)

MRI: Magnetic resonance imaging; EEG: Electroencephalogram; FIRDA/TIRDA: Frontal or temporal intermittent rhythmic delta activity.

Routine CSF examination was normal in 10 patients (32.3%) and revealed lymphocytic meningitis in 67.7% of cases with an average of white blood cells of 54/µl (range 19–140), an average of proteinorachia of 0.56 g/L (range 0.23–1.37) and an average of glycorrhachia of 0.54 g/L (range 0.34–0.71). Serological tests for syphilis in the CSF had been performed in 30 patients and were positive in five cases. The patient presenting a vesicular rash that occurred 7 days before its admission to our Department (the sole patient who had not benefited from serological tests for syphilis), diagnosis of chickenpox was suspected. The serological tests revealed high plasma levels of IgM specific for VZV (varicella‐zoster virus). The CSF examination in this patient revealed a white blood cell count of 54/µl, with glucose of 0.67 g/L and protein of 0.42 g/L. Although VZV‐PCR has not been performed in the CSF, diagnosis of varicella LE was considered. We performed HSV‐PCR in the CSF in only 14 patients (41.2%), of whom it was positive in 2 cases.

The systemic immunological tests and thyroid function tests were performed in 11 cases and were negative. The measurement of antineuronal antibodies has been performed in seven patients, and antibodies anti‐NMDA‐R were positive in two cases. In these patients with anti‐NMDA‐R LE, thoracic‐abdominopelvic CT scan and pelvic ultrasound were normal. Seven other patients had benefited from thoracic‐abdominopelvic CT scan in whom it revealed lung cancer in two cases, ovarian teratoma in one case (already known in this patient), mediastinal and hilar lymphadenopathies without parenchymal lung lesions in one case, and normal findings in three cases. The diagnosis of paraneoplastic autoimmune LE was considered in these patients with lung cancer and ovarian teratoma although antineuronal antibodies were negative. The conditions that led to the diagnosis of paraneoplastic autoimmune LE were the presence of existing cancer, the subacute onset of symptoms, bilateral and symmetrical MRI abnormalities on FLAIR sequences and T2‐weighted images in the medial temporal lobes, and EEG abnormalities in the temporal regions. These conditions meet the diagnostic criteria of Graus et al (2016) for the diagnosis of autoimmune LE. The thyroid function tests were also normal in these three patients with lung cancer and ovarian teratoma. The diagnosis of paraneoplastic autoimmune LE was also considered in four others patients among which two known with cancer of the cavum, one with melanoma of the thigh, and one patient with thyroid cancer. The measurement of antineuronal antibodies was made only in one of these last four patients. The thyroid function tests were normal in these four patients. Overall, we considered the diagnosis of paraneoplastic autoimmune LE in seven patients and anti‐NMDA‐R LE in two patients.

In one patient, we made the diagnosis of sarcoidosis LE whose observation was previously published (Toudou‐Daouda, Assadeck, & Efared, 2017). The diagnosis of sarcoidosis LE has been confirmed by the histological examination of nasal biopsy that showed a granulomatous inflammation made of confluent granulomas with multinucleated giant and epithelioid cells surrounded by a rim of lymphocytes without caseous necrosis. In this patient, the thoracic‐abdominopelvic CT scan revealed mediastinal and hilar lymphadenopathies without parenchymal lung lesions. In addition, the dosage of angiotensin‐converting enzyme revealed high plasma levels. The MRI abnormalities in this patient were hyperintensities in right temporo‐insular region on FLAIR sequences and T2‐weighted images and on gadolinium‐enhanced T1‐weighted images, an enhancement and nodular leptomeningeal thickening in the basilar perimesencephalic cistern extended to the right temporal lobe, hypothalamus, and third ventricle. We also made the diagnosis of tuberculous LE in one patient whose observation was previously published (Toudou Daouda, Obenda, & Souirti, 2017). The diagnosis of tuberculous LE was considered according to the diagnostic criteria of the consensus case definitions for tuberculous meningitis (Marais et al., 2010). The MRI abnormalities in this patient were a bilateral basifrontal and mesiotemporal hyperintensities on FLAIR sequences interesting the right insula, associated with nodular contrast enhancement of these same lesions and an enhancement and nodular leptomeningeal thickening of the external part of the right temporal lobe on gadolinium‐enhanced T1‐weighted images. The thoracic‐abdominopelvic CT scan was normal. Systemic immunological tests and the measurement of antineuronal antibodies were negative, as well as the HSV‐PCR.

Overall, the different etiologies are syphilis (five cases), varicella (one case), HSV (two cases), tuberculosis (one case), sarcoidosis (one case), anti‐NMDA‐R LE (two cases), and paraneoplastic autoimmune LE (seven cases). We found 12 cases of LE without definite etiology and with an incomplete diagnostic workup. The systemic immunological tests and the measurement of antineuronal antibodies had not been performed in all these 12 patients, as well as thoracic‐abdominopelvic CT scan. Only six of the 12 patients had benefited from HSV‐PCR, which was negative. Serological tests for syphilis in the CSF were negative in the 12 patients. The thyroid function tests were performed in only two of the 12 patients and were negative.

For the patients with paraneoplastic autoimmune LE, radiotherapy has been main cancer treatment coupled with chemotherapy in some patients. Only the patient with melanoma of the thigh had benefited a surgical treatment coupled with chemotherapy.

The medium‐term clinical course includes a complete remission in 45.2% of cases and partial remission in 45.1% of cases. The different sequelae were temporal lobe epilepsy (9.7%), anterograde amnesia (16.1%), and severe cognitive impairment (19.4%). Three deaths (9.7%) had been registered in our study. One patient died from severe brain injuries of LE. The other patients died because of other associated diseases. The patient with melanoma of the thigh died from a massive pulmonary embolism. The patient with ovarian teratoma died 5 months after the diagnosis of LE by complications of the ovarian teratoma.

## 4 DISCUSSION

In this retrospective study of 31 Moroccan patients diagnosed with LE in the Department of Neurology of the Hassan II University Teaching Hospital of Fez (Morocco) between September 2008 and December 2016, we described the demographic, clinical, paraclinical, and etiological features of LE, as well as its medium‐term prognosis. In the end, we found not only a male sex predominance (71%) but also a high frequency of paraneoplastic autoimmune LE (22.6%) and a predominance of syphilis among the infectious causes (5/9 cases; 55.6%). In addition, we found a hospital frequency of LE of 0.81%.

The clinical manifestations of LE in our study were polymorphic, but very typical of those reported in the literature: acute or subacute onset of anterograde memory disorders, psychiatric disorders, confusional state, temporal lobe, or generalized epilepsy (Anderson, & Barber, 2008; Corsellis et al., 1968; Geisler et al., 2013; Kerling et al., 2008). Epileptic seizures were the most common clinical manifestations in our study (54.8%), which is consistent with the literature data (Aupy et al., 2013; Irani, Bien, & Lang, 2011; Irani, Michell, et al., 2011; Vincent et al., 2004). In the literature, the clinical picture is essentially subacute (Alamowitch et al., 1997; Corsellis et al., 1968; Gultekin et al., 2000; Lawn, Westmoreland, Kiely, Lennon, & Vernino, 2003; Vedeler et al., 2006), whereas in our study, the onset was acute (1–7 days) in 64.5% of cases. This could be explained by the fact that infectious causes are more common in our study because these etiologies usually have an acute onset of symptoms (Geisler et al., 2013; Hirai et al., 2005). The autoimmune LE most often has a subacute onset of symptoms.

Functional cerebral imaging (SPECT or FDG‐PET) is the most sensitive radiological examination in the diagnosis of LE allowing to highlight signs of hypermetabolism in the temporal and extratemporal regions (Franck et al., 1987 *;* Provenzale, Barboriak, & Coleman, 1998; Fakhoury, Abou‐Khalil, Kessler, 1999; Na, Hahm, Park, & Kim, 2001; Kassubek et al., 2006). This examination was not performed in our study because it is not available in our hospital, and its practice was not indispensable because all patients had positive brain MRI. EEG is a frequently performed examination in cases of suspected LE, but its practice is not required to make the diagnosis of LE. The EEG abnormalities commonly reported are diffuse slowing without epileptiform activity, slowing of the background rhythm, epileptiform activity in the temporal or frontotemporal regions, periodic lateralized epileptiform discharges in the temporal regions (Kerling et al., 2008 *;* Anderson, & Barber, 2008 *;* Asztely, & Kumlien, 2012 *;* Franck et al., 1987; Fakhoury, Abou‐Khalil, Kessler, 1999; Scheid, Lincke, Voltz, Von Cramon, & Sabri, 2004 *;* Ances et al., 2005). In our study, the main EEG abnormalities included the slow waves in the temporal regions, slowing of the background rhythm, spikes, and waves in the frontotemporal regions.

We found in this study 12 cases (38.7%) of LE without definite etiology, but with an incomplete diagnostic workup. We think that among these patients, some of them could have probably diagnosis of herpetic LE because of the acute onset of symptoms and the clinical improvement under antiherpetic treatment, and knowing that only six of these patients had benefited from HSV‐PCR in CSF. On the other hand, these patients may simply correspond to patients who spontaneously improve and who would have had a favorable evolution without antiherpetic treatment.

Of the infectious causes of LE, HSV is the most common infectious agent (Asztely, & Kumlien, 2012). Other infectious causes are also reported such as syphilis, VZV, and more rarely tuberculosis. The case of tuberculous LE reported in our study is the second case reported in the literature to our knowledge after that reported by Sonkaya et al. (2014).

The paraneoplastic autoimmune LE seems to be the most common causes of LE in our study (7/31 cases; 22.6%). The diagnosis of paraneoplastic autoimmune LE was made in our patients according to the diagnostic criteria for autoimmune LE of Graus et al. (2016) which are (a) subacute onset (rapid progression of <3 months) of working memory deficits, seizures, or psychiatric symptoms suggesting involvement of the limbic system, (b) bilateral brain abnormalities on T2‐weighted FLAIR MRI highly restricted to the medial temporal lobes, (c) CSF pleocytosis (white blood cell count of more than five cells per mm^3^) or EEG with epileptic or slow‐wave activity involving the temporal lobes, and (d) reasonable exclusion of alternative causes. When the four criteria mentioned above are met, the positivity of antineuronal antibodies is not indispensable to consider LE as having a definite autoimmune origin. In addition, studies have shown that autoimmune LE can occur without detectable autoantibodies (Graus et al., 2008; Najjar, Pearlman, Zagzag, & Devinsky, 2011). However, the detection of antineuronal antibodies may be important to determine the immunological type of LE, to guide to an associated tumor, and to evaluate the prognosis which might differ according to the type of antibody (Graus et al., 2016; Höftberger et al., 2015; Malter et al., 2014). On the other hand, if one of the first three criteria above mentioned is not met, the diagnosis of autoimmune LE can also be made when the antineuronal antibodies are positive (Graus et al., 2016).

Anti‐NMDA‐R encephalitis affects mainly women, and the clinical manifestations included abnormal behavior, memory disorders, speech disorders, epileptic seizures, abnormal movements (orofacial, limb, or trunk dyskinesias), loss of consciousness or autonomic dysfunction, central hypoventilation, and cerebellar ataxia or hemiparesis (Titulaer et al., 2013). Our two patients with anti‐NMDA‐R LE were women. The clinical manifestations were characterized by temporal lobe epilepsy, abnormal behavior, memory disorders and confusional syndrome in one patient, and epileptic seizures associated with abnormal behavior and language disorders in the other patient. One patient had anterograde amnesia as sequelae, while the other patient recovered completely. A study showed that the patients with anti‐NMDA‐R LE have a better prognosis when a tumor is discovered and resected than the patients without any tumor found (Florance et al., 2009). The complete remission in our patient with anti‐NMDA‐R LE may simply correspond to a spontaneously favorable evolution, and the immunomodulatory treatment would not have been necessary to permit de complete remission as previously described in the literature (Iizuka et al., 2008).

Our study has some limitations. First, the diagnostic tools of LE are poorly developed in our hospital in particular and in Morocco in general: (a) lack of functional imaging (SPECT and FDG‐PET) not only for the positive diagnosis of LE but also for research of associated cancer (FDG‐PET/CT full body), (b) limited access to systemic immunological tests, antineuronal antibodies, and HSV‐PCR. Secondarily, our study had not included patients with negative brain MRI because of the diagnostic criteria for autoimmune LE of Graus et al. (2016) are not met in these patients. In addition, there is no evidence of a particular infectious cause especially herpes infection. Thus, it is possible that a good part of the patients with autoimmune LE with negative brain MRI or patients with benign herpetic LE who had not benefited from HSV‐PCR in the CSF has not included in this study. Thirdly, the retrospective nature of this study explains why some details were not provided especially some clinical data and treatments.

## 5 CONCLUSION

Our study demonstrates a wide diversity of etiologies of LE in Morocco with essentially an acute mode of onset of symptoms suggesting a probable predominance of infectious causes because some patients with LE without definite etiology had responded well to antiherpetic treatment. However, the frequency of patients with definite autoimmune LE is similar to that of patients with definite infectious LE (29% in both groups). The large number of patients with an incomplete diagnostic workup demonstrates that adequate laboratory tests are needed in Morocco to improve the management of the patients with LE.

## CONFLICT OF INTERESTS

The authors declared no potential conflicts of interest with respect to the research, authorship, and/or publication of this article.

## AUTHORS’ CONTRIBUTIONS

AFA and AS conceived and designed the study, collected and analyzed the data. MTD wrote the first draft of the manuscript in its entirety and participated to the interpretation of the MRI and EEG. MFB and ZS participated in the design and conception of the study, interpreted MRI and EEG, and had critically revised the manuscript. All authors have seen and approved the final version of the manuscript.
